# Impact of fertility treatments on headache disorders: a systematic review with an overview of treatment modalities

**DOI:** 10.1186/s10194-026-02325-y

**Published:** 2026-03-24

**Authors:** Carolin Luisa Hoehne, Alina Lohner, Yones Salim, Maria Terhart, Wiebke Andersen, Cornelius Angerhöfer, Rüdiger Moltrecht, Florian Scheuerecker, Antoinette Maassen van den Brink, Bianca Raffaelli

**Affiliations:** 1 Department of Neurology with Experimental Neurology, Charité-Universitätsmedizin Berlin, Corporate Member of Freie Universität Berlin and Humboldt-Universität zu Berlin, Berlin, Germany; 2 Department of Gynaecology, University Medical Center Hamburg-Eppendorf, Hamburg, Germany; 3 Kinderwunschzentrum Göttingen, Center for Reproductive Medicine, Göttingen, Germany; 4 Division of Pharmacology and Vascular Medicine, Department of Internal Medicine, Erasmus University Medical Center Rotterdam, Rotterdam, Netherlands; 5 Clinician Scientist Program, Berlin Institute of Health (BIH), Berlin, Germany

**Keywords:** Fertility treatment, Migraine, Headache, IVF

## Abstract

**Introduction:**

Infertility represents a global health concern and assisted reproductive technologies expose an increasing number of women and men to intensive hormonal interventions. At the same time, migraine and other headache disorders are highly prevalent in the reproductive age group and hormonal fluctuations are well-established headache triggers. The impact of fertility treatments on headache remains unclear and guidance for clinical management is lacking.

**Methods:**

This systematic review followed PRISMA guidelines and was pre-registered in PROSPERO. PubMed and Embase were searched up to 1^st^ of August 2025 using predefined strategies combining “migraine” or “headache” with fertility-related terms. Original human studies reporting on common fertility treatments and headache were included. Data were synthesized qualitatively due to heterogeneity in design and outcomes. In parallel, headache-related adverse events were extracted from Food and Drug Administration or, alternatively, European Medicines Agency drug labeling for fertility medications.

**Results:**

The search retrieved 3,305 unique records, of which 196 full-text articles were assessed and 104 met inclusion criteria. Across studies of selective estrogen receptor modulators, aromatase inhibitors, gonadotropins, analogous of gonadotropin-releasing hormone, and luteal phase support with progesterone or estrogen, headache was frequently reported, with rates ranging from rare (<1%) to nearly 50%, depending on agent and protocol. Reporting was typically nonspecific, with almost no information on headache type, severity, or temporal relation to treatment phases. Case reports and a retrospective case–control study linked headache to treatment-related complications such as ovarian hyperstimulation syndrome (OHSS) and cerebrovascular events, with migraine history associated with a markedly increased risk for OHSS.

**Conclusion:**

Headache is a common but undercharacterized adverse event across fertility treatments. Given the potential contribution of hormonal, vascular, and psychosocial mechanisms, systematic assessment of headache, careful evaluation of patients with migraine or vascular risk factors, and standardized reporting in future trials are essential to inform individualized and safer reproductive care.

**Clinical trial number:**

Not applicable.

## Introduction

Infertility is a significant and growing global health concern, affecting an estimated 110 million women and 55 million men worldwide as of 2021 [1, 2]. The burden of infertility is highest among women aged 35–39 years, and the prevalence has increased by over 80% since 1990, with projections indicating a continuing rise through 2040 [1, 2]. Infertility is associated with substantial psychological distress, including increased rates of depression and anxiety, and can have profound social and economic consequences for affected individuals and couples [3]. The etiology of infertility is multifactorial, encompassing endocrine, anatomical, genetic, and environmental factors, and remains unexplained in a significant proportion of cases [4].

In response to the rising prevalence and impact of infertility, the use of assisted reproductive technologies (ART), particularly in vitro fertilization (IVF), has expanded rapidly worldwide. Globally, more than 3.5 million ART cycles are performed each year, resulting in almost one million born infants [5, 6]. While ART has improved reproductive outcomes for many, it also introduces new physiological and psychosocial challenges, including the potential for adverse effects on neurological health [7, 8]. In addition to fertility treatment, ART is increasingly used for fertility preservation through oocyte or embryo freezing, both in medical contexts, such as prior to gonadotoxic cancer therapy, and for social reasons, contributing to a broader and growing population undergoing hormonal stimulation [9]. The complexity of ART regimens, the emotional burden of infertility, and the uncertainty of outcomes can contribute to psychological distress and may impact quality of life.

The relationship between headache disorders, most notably migraine, and fertility is increasingly recognized as bidirectional [10]. Women with migraine are more likely to avoid or delay pregnancy due to concerns about symptom exacerbation, medication teratogenicity, and disability during gestation, with up to 20% of women reporting avoidance of pregnancy for these reasons [11]. Notably, these concerns contrast with clinical observations showing that migraine improves during pregnancy in approximately 60–80% of women [12]. In addition, pre-existing migraine is associated with a higher prevalence of endocrine comorbidities such as endometriosis or thyroid disorders, which may impair fertility [13, 14]. Within the spectrum of endocrine disorders affecting fertility, polycystic ovary syndrome (PCOS) occupies a central role, as it is a leading cause of anovulatory subfertility and a frequent indication for fertility treatment [15]. In women with PCOS, the relationship with migraine is complex and characterized by heterogeneous findings. While some studies report an increased prevalence of migraine in women with PCOS, others found no association or even suggested a reduced prevalence, potentially related to attenuated cyclical hormonal fluctuations [13, 16, 17].

Fertility treatments themselves may provoke or worsen headache, particularly in women with a prior history of migraine. Despite the clinical relevance and growing use of fertility treatments, evidence on the occurrence, characteristics, and clinical context of headache during fertility treatment remains limited and heterogeneous. To address this gap, we present a systematic review of the available literature on whether fertility treatments may trigger or exacerbate headache.

## Methods

This review was conducted in accordance with the Preferred Reporting Items for Systematic Reviews and Meta-Analyses (PRISMA) guidelines.

The review protocol was pre-registered in PROSPERO 2025 (CRD420251119054). After the abstract screening stage, the scope of the review was refined to focus exclusively on studies examining common fertility treatments and headache.

The primary objective of this systematic review was to examine whether fertility treatments have an impact on headache, both in individuals with pre-existing headache disorders and in those without a prior headache history.

Given the heterogeneity of study designs, populations, treatments and outcome measures, a quantitative meta-analysis was not feasible, and findings were synthesized in a systematic review.

### Screening and Selection

A comprehensive literature search was performed in PubMed and Embase from database inception up to August 2025. The PubMed search (conducted on August 1, 2025) used the terms (migraine OR headache) AND (fertil* OR infertil*). The Embase search was performed on August 15, 2025, using the following strategy:

exp migraine/OR migraine.ti,ab. OR exp headache/OR headache*.ti,ab.*; exp fertility/OR fertil.ti,ab. OR exp infertility/OR infertil*.ti,ab.*; 1 AND 2.

In response to peer-review comments and to improve sensitivity, the PubMed search strategy was subsequently expanded on the 8th of February 2026 to include commonly used treatment-related terms referring to assisted reproductive technologies. We used the following terms “IVF”, “ICSI”, “ovarian stimulation”, “OHSS”, embryo transfer”, “luteal support”, “cryopreservation”, “agents, fertility[MeSH Terms]”, each combined with “migraine OR headache”. Duplicates were removed using EndNote 2025.

All retrieved records underwent a two-stage screening process. In the first stage, titles and abstracts were screened by independent reviewers. Six reviewers (CLH, CA, MT, WA, YS, BR) participated in this process, and each record was evaluated by two reviewers randomly assigned within this pool. Any record judged as potentially eligible by either reviewer proceeded to full-text screening. In the second stage, full-text articles were reviewed independently by two reviewers (CLH, BR) against predefined inclusion and exclusion criteria. Discrepancies at either stage were resolved through discussion, and a third reviewer was consulted if consensus could not be reached.

#### Inclusion and Exclusion Criteria

Studies were considered eligible for inclusion if they were original research articles that reported on common fertility treatment (such as selective estrogen receptor modulator (SERM)), aromatase inhibitors, gonadotropins, gonadotropin-releasing hormone(GnRH)-analogue and luteal support with estrogens or progesterone) and headache, either pre-existing or newly developed. Only full-text articles published in English were included. Eligible study designs comprised randomized controlled trials, prospective or retrospective observational studies, and registry-based analyses.

We excluded publications (1) that were not original research, such as reviews, conference abstracts, posters or letters. (2) Studies that did not involve human participants or (3) that lacked relevant outcome data on headache in the context of fertility treatment were also excluded. (4) Fertility treatment with other substances (like metformin), adjunctive or additive treatment, was also excluded. (5) We excluded articles focusing solely on hyperprolactinemia, which is often caused by pituitary tumors, even though both can cause headaches, contribute to infertility, or the tumor can enlarge during fertility treatment [18]. Given the extensive literature on this topic and its classification as a secondary headache, we did not include it in our analysis [19, 20].

### Data Extraction and Analysis

Data from eligible studies were extracted into a standardized table, including study design, type of fertility treatment, reason for fertility treatment, number of study population and headache outcomes. We grouped fertility treatments by drug class and sex.

### Drug-Label

In addition to the systematic literature review, information on reported headache as an adverse event was extracted from the official U.S. Food and Drug Administration (FDA) drug labeling database. For all pharmacological agents used in fertility treatment identified in this review, the most recent FDA-approved prescribing information was accessed via the FDA website on October 29, 2025. Data were extracted for each drug regarding the presence, frequency, and classification of headache within the adverse reactions or postmarketing experience sections.

When FDA labeling was unavailable, data were retrieved from the European Medicines Agency (EMA) product information database.

## Overview of Fertility Treatments

Infertility is defined as the inability to achieve pregnancy after 12 months of regular, unprotected sexual intercourse [4]. Fertility evaluation and treatment is indicated after this time, even earlier in certain cases (for example, in advanced maternal age, or with known risk factors). Around 30–40% of cases are attributed to female factors, 30–40% to male factors, and in 20–30% both partners contribute [21].

**Fig. 1:**
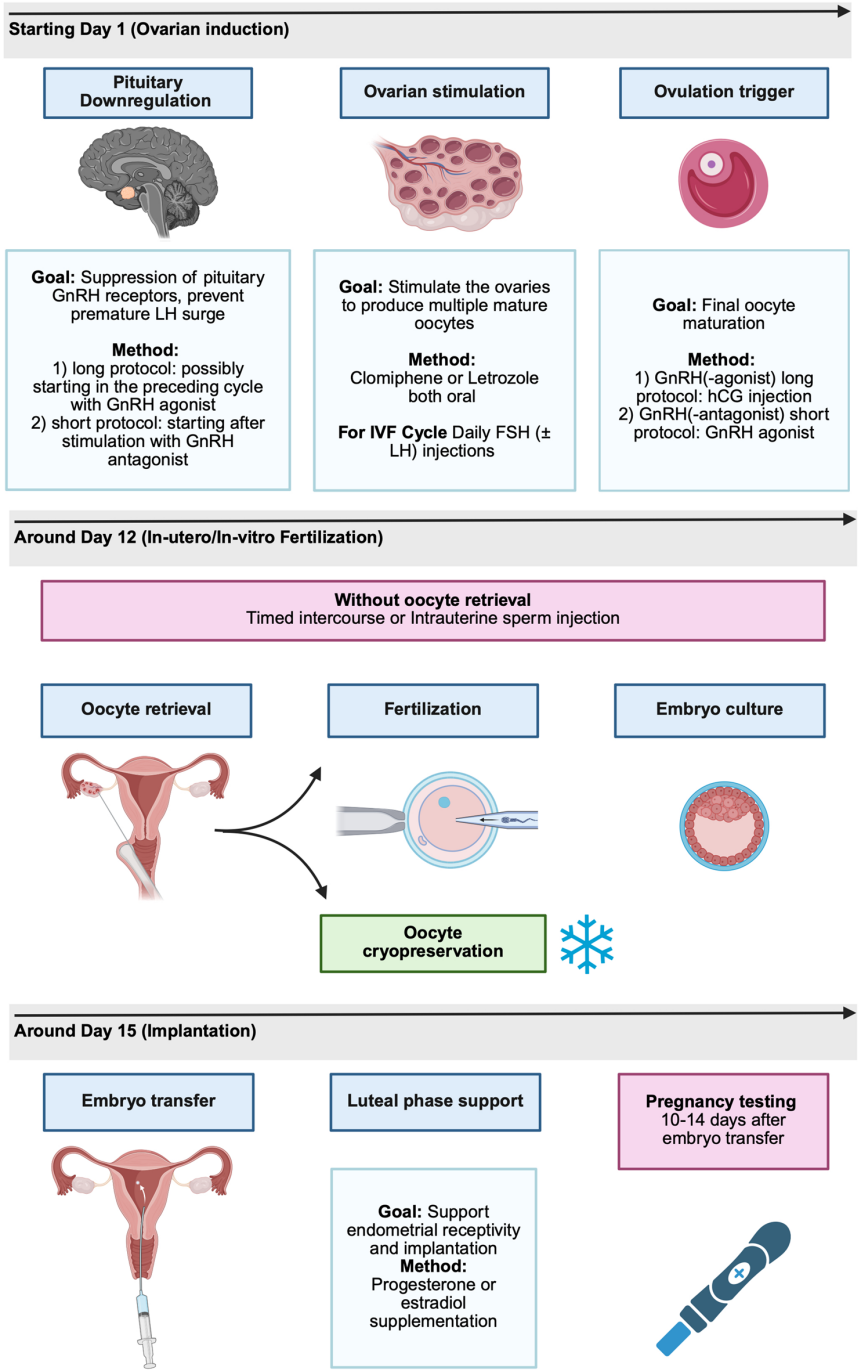
Overview of fertility treatment. Overview of a standard fertility treatments, illustrating key hormonal intervention phases from ovarian induction and stimulation to fertilization, embryo transfer, and luteal phase support. Created with BioRender.com

To contextualize the findings of this systematic review and support the interpretation of the included studies, we provide a brief overview of fertility treatment strategies (Fig. 1). These include ovulation induction alone or in combination with intrauterine insemination (IUI), as well as more advanced techniques such as IVF and intracytoplasmic sperm injection (ICSI). This section is intended solely as clinical background information to aid interpretation of the results and does not represent part of the systematic review methodology.

### Ovulation Induction and Ovarian Stimulation

Ovulation induction and ovarian stimulation are among the main treatment approaches in fertility treatment, with the first aiming to trigger ovulation in anovulatory women, and the second helping to stimulate the development of multiple follicles, also in normally ovulating women [22].

Two main oral agents are mostly used: clomiphene (or clomiphene citrate) and letrozole [4]. Clomiphene is a SERM that blocks estrogen receptors in the hypothalamus, thereby disrupting the normal negative feedback mechanism [23]. This leads to an increased release of GnRH, which subsequently stimulates follicle-stimulating hormone (FSH) and luteinizing hormone (LH), promoting follicular growth and ovulation. Clomiphene has been the first-line treatment for anovulatory infertility, with ovulation rates of up to 80% and pregnancy rates of approximately 30–40% over multiple cycles [22].

Letrozole inhibits the aromatase enzyme responsible for converting androgens to estrogens, thereby reducing circulating estrogen levels. The resulting decrease in negative feedback to the hypothalamus enhances GnRH secretion and promotes FSH release. Recent trials show that letrozole achieves higher live birth rates than clomiphene, especially in overweight or clomiphene-resistant women, and is now the preferred first-line therapy for PCOS [24].

In cases where oral treatments are ineffective, or when higher follicular recruitment is desired such as in IVF and ICSI cycles, exogenous gonadotropins (recombinant FSH, with or without LH, or human menopausal gonadotropin (hMG) can be administered for ovarian stimulation [25]. These injectables directly stimulate follicular development and are more potent than oral medications. However, they carry a significantly higher risk of multiple gestation and ovarian hyperstimulation syndrome (OHSS), a potentially serious iatrogenic complication characterized by increased vascular permeability, ascites, and thromboembolic risk [4]. Gonadotropin protocols require careful monitoring with serial transvaginal ultrasound and serum estradiol levels measurements to optimize response and minimize complications.

Ovulation induction and ovarian stimulation can be used in combination with timed intercourse, IUI, IVF or ICSI.

### Intrauterine Insemination

IUI involves the direct injection of motile sperm into the uterine cavity, bypassing the cervical barrier and increasing the chances of fertilization. IUI is usually timed 24 to 36 hours after administration of an ovulation trigger. IUI solely and without ovulation induction is not recommended because it does not improve pregnancy rates in uncertain infertility [4].

### In-Vitro Fertilization, Intracytoplasmic Sperm Injection and Cryopreservation

When less invasive treatments are unsuccessful, or in the presence of specific indications such as severe male factor infertility or tubal obstruction, ovarian stimulation followed by transvaginal oocyte retrieval is performed for IVF or ICSI. Oocyte retrieval is also carried out for fertility preservation, either for medical reasons (e.g., prior to gonadotoxic therapy) or increasingly for elective purposes to preserve reproductive potential with advancing age [26, 27].

This process involves multiple coordinated phases [28]. Ovarian stimulation is preceded or accompanied by pituitary downregulation, which can be achieved using either a long GnRH agonist protocol or a short GnRH antagonist protocol [29].

In the long GnRH agonist protocol, treatment is initiated in the preceding menstrual cycle, prior to the stimulation cycle. Administration of a GnRH agonist initially induces a transient flare effect, characterized by a short-term increase in LH secretion [29]. With continued administration, pituitary GnRH receptors become downregulated, leading to suppression of endogenous gonadotropin release and reduced estradiol levels. This establishes a stable hormonal baseline prior to controlled ovarian stimulation with exogenous gonadotropins.

In contrast, the short (antagonist) protocol involves the administration of a GnRH antagonist during the mid-follicular phase of ovarian stimulation, providing immediate suppression of endogenous LH secretion and preventing a premature LH surge without an initial flare effect [29].

Follicular development is monitored through serial transvaginal ultrasound examinations and hormonal assays. Once the leading follicles reach an appropriate diameter, final oocyte maturation is induced by a trigger injection using either human chorionic gonadotropin (hCG) in the long protocol or a short-acting GnRH agonist in the short protocol. This trigger mimics the natural LH surge and is precisely timed to allow oocyte retrieval approximately 34–36 hours later, before spontaneous ovulation occurs.

Transvaginal oocyte retrieval is performed under ultrasound guidance and light sedation or general anesthesia. A needle is inserted into each follicle to aspirate follicular fluid, from which mature oocytes are isolated in the embryology laboratory. On the same day, sperm cells are collected and fertilization is performed. In conventional IVF, thousands of sperm cells are co-incubated with each mature oocyte. Alternatively, ICSI may be used, especially in cases of severe male factor infertility. ICSI involves the direct injection of a single sperm cell into the oocyte cytoplasm, significantly increasing the chance of fertilization [30].

Fertilized embryos are cultured in-vitro for several days, normally for 2–6 days. At this point, one or more embryos are selected for transvaginal embryo transfer into the uterine cavity using a thin catheter. The luteal phase is supported with progesterone or estogene supplementation to enhance the implantation potential [31].

Approximately 10–14 days after embryo transfer, a serum β-hCG test is performed to confirm pregnancy. If positive, a transvaginal ultrasound is scheduled at around 6–7 weeks of gestation to confirm intrauterine implantation and detect fetal cardiac activity.

For oocyte cryopreservation, oocytes are retrieved following ovarian stimulation, either as part of elective fertility preservation or when oocytes obtained during IVF or ICSI cycles are not used in the cycle. They are then preserved via vitrification, a rapid-freezing technique that maintains oocyte integrity by minimizing ice crystal formation.

### Male Infertility

Depending on the underlying etiology, male infertility may be managed with medical therapy, surgical interventions, or assisted reproductive technologies [32]. Many infertile men present with primary testicular dysfunction, characterized by impaired spermatogenesis and reduced sperm output. These conditions are typically associated with hypergonadotropic hypogonadism, reflecting a lack of negative feedback from the testes despite increased pituitary gonadotropin secretion. As the target organ, the testes, does not respond adequately, causal treatment options are limited, and most affected men require assisted reproductive technologies such as IVF or ICSI to achieve conception [32]. In a small subset of men, primarily those with hypogonadotropic hypogonadism, infertility can be treated causally, as the hypothalamic–pituitary–testicular axis is intact and spermatogenesis can be stimulated through gonadotropin therapy [33]. In these cases, treatment with hCG with or without FSH represents the established first-line approach.

For idiopathic infertility or oligozoospermia with low testosterone, SERMs such as clomiphene or aromatase inhibitors may be used off-label, although supporting evidence remains limited [34]. Importantly, exogenous testosterone is contraindicated, as it suppresses spermatogenesis [35].

## Results: Does Fertility Treatment Have an Impact on Headache?

**Fig. 2:**
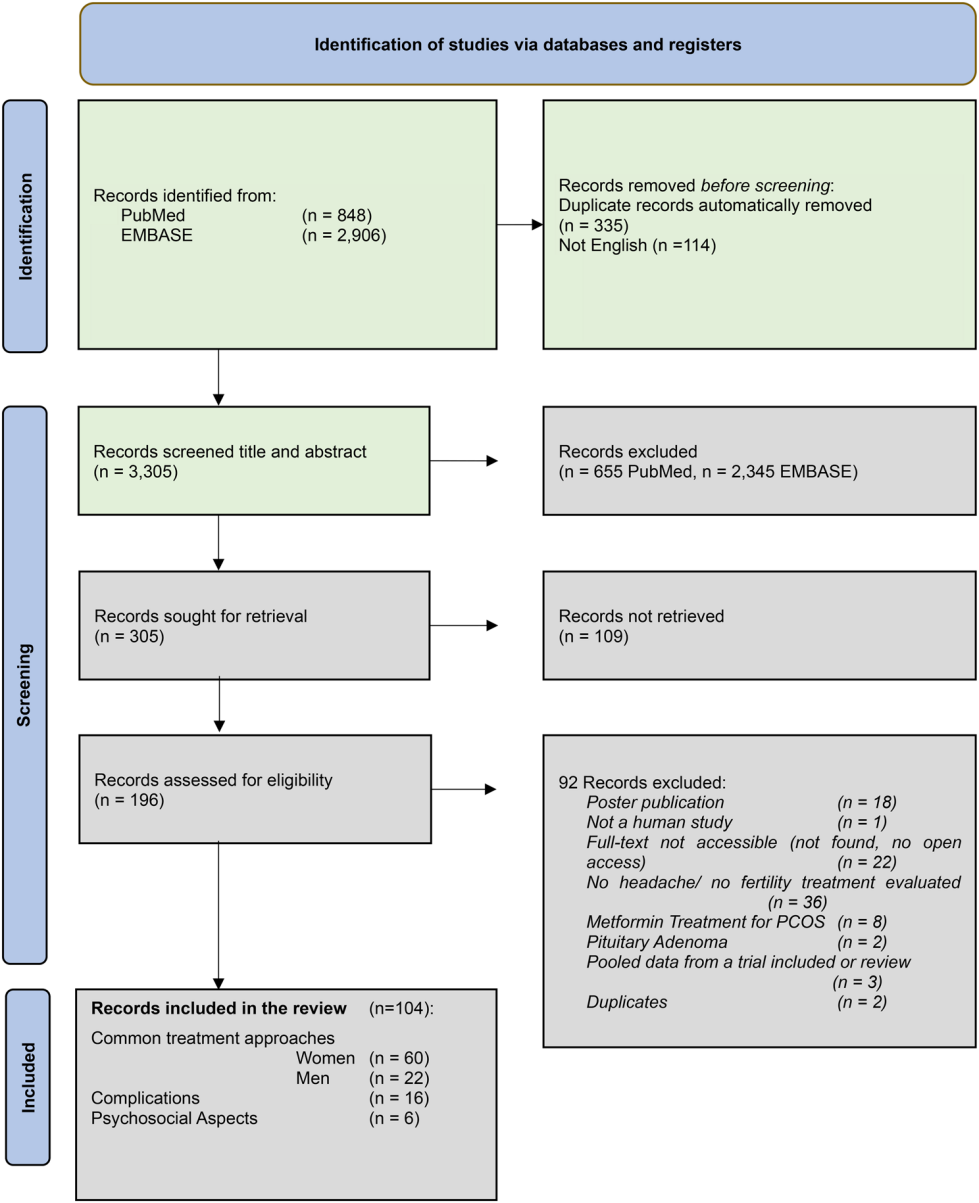
PRISMA flow diagram of study selection

This systematic review identified studies addressing the relationship between fertility treatment and headache, both in individuals with and without pre-existing headache disorders. The initial literature search retrieved 848 records from PubMed and 2,906 from Embase, published up to August 2025 (Fig. 2). After removal of duplicates, 3,305 records remained for title and abstract screening. Of these, 196 articles were selected for full-text review, and 92 were excluded based on predefined criteria. To provide a structured overview, results are organized into thematic areas: (1) common fertility treatment approaches for both women and men (Tables 1, 2, 3 and 4), (2) treatment-related complications (Table 5), and (3) psychosocial aspects. To contextualize the literature findings, we first summarize the reported rates of headache and migraine in the FDA and/or EMA labels of key medications used in fertility treatment. We then present the results of the included studies identified through our systematic search.

### Common Fertility Treatment Approaches

#### Selective Estrogen Receptor Modulators and Aromatase Inhibitors

According to FDA labeling, SERM show a lower incidence of headache (1.3–9.2%) compared with aromatase inhibitors (8–13.1%) [36–40]. Migraine as a side effect is explicitly mentioned for the SERMs clomiphene and raloxifene.

Consistent with these safety data, many studies retrieved in our search have also reported headache as a frequent adverse event during oral fertility treatment (Table 1). However, the included studies varied in design and size, comprising 17 randomized controlled trials and 6 observational studies, with sample sizes ranging from *n* = 15 to *n* = 271. Reporting of headache was inconsistent across studies, and there was no information about intensity or type of headache. Migraine was only once mentioned as a side effect of clomiphene [41].

**Table 1:** Selective estrogen receptor modulators and aromatase inhibitors and headache reports. PCOS: polycystic ovary syndrome

**Medication Group**		**Drug**	**Clinical Trials**	**Post-marketing Research** **(Central Nervous System)**		**FDA-Label Report Link**					
Selective Estrogen Receptor Modulator		Clomiphene	Headache 1.3%	Migraine headache, paresthesia, seizure, stroke, syncope		https://www.accessdata.fda.gov/drugsatfda_docs/label/2012/016131s026lbl.pdf					
		Tamoxifen	n/a	less frequently reported headaches		https://www.accessdata.fda.gov/drugsatfda_docs/label/2005/17970s053lbl.pdf					
		Raloxifene	Headache 9.2%	Migraine headache, vertigo, neuralgia, hypesthesia		https://www.accessdata.fda.gov/drugsatfda_docs/label/2007/020815s018lbl.pdf					
Aromatase Inhibitor		Letrozole (Femara)	Headache 8%	Headache, dizziness, insomnia		https://www.accessdata.fda.gov/drugsatfda_docs/label/2014/020726s027lbl.pdf					
		Anastrozole	Headache 8–10%	Headache, dizziness,insomnia, anxiety, paresthesia, depression		https://www.accessdata.fda.gov/drugsatfda_docs/label/2011/020541s026lbl.pdf					
**WOMEN**											
**Author, Year**	**Study Design**		**Reason for Subfertility**	**Fertility Treatment and Protocol**	**Study population (N)**	**Group 1 (n)**	**Group 2 (n)**	**Group 3 (n)**	**%** **headache in group 1**	**% headache in group 2**	**%** **headache in group 3**
(Tredway et al. 2004) [42]	Phase I clinical trial		Healthy, premenopausal female volunteers	Anastrozole (single dose vs. five daily doses)	26	20	6	n/a	36.0% of all subjects (no dose dependency, not specified)		
(Li et al. 2025) [43]	Open-label, randomized trial		PCOS	Letrozole (2.5 mg vs 5 mg)	174	87	87	n/a	18%	18%	n/a
(Jahan et al. 2022) [44]	Open-label, randomized trial		PCOS	Letrozole (low-dose extended vs double-dose short protocol, each 3 cycles)	66	34	32	n/a	10%	5%	n/a
(Amer et al. 2017) [41]	Double-blind randomized trial		PCOS	Letrozole vs Clomiphene	159	79	80	n/a	Headache in letrozole group, migraine in clomiphen group (no specification)		
(Thomas et al. 2019) [45]	Retrospective cohort study		PCOS	Letrozole vs Clomiphene	92	49	43	n/a	“A larger proportion of women in the CC group reported having experienced any side effects associated with treatment than the Letrozole group (41.9% vs 8.2%, *p* < 0.001). The side effects reported include bone/muscle pain, climacteric, headaches, gastrointestinal and fatigue. “		
(Al-Thuwaynee & Swadi, 2023) [46]	Open-label, randomized trial		PCOS	Letrozole vs Clomiphene	100	50	50	n/a	4%	6%	n/a
(Chera-Aree et al. 2023) [47]	Randomized, controlled trial		Ovulation Dysfunction	Letrozole + Clomiphene vs Clomiphene	100	50	50	n/a	13%	5%	n/a
(Mejia et al. 2019) [48]	Open-label, randomized trial		PCOS	Letrozole + Clomiphene vs Letrozole	70	35	35	n/a	28%	41%	n/a
(Panda et al. 2023) [49]	Triple-blind, placebo-controlled trial		PCOS	Letrozole + Clomiphene vs Letrozole	80	40	40	n/a	26%	29%	n/a
(Von Wolff et al. 2014) [50]	direct comparitive trial		Different reasons	HCG vs HCG + clomiphene	112	108	103	n/a	0%	5%	n/a
(Jones et al. 2018) [51]	Retrospective cohort study		PCOS	Clomiphene (two different protocols)	109	66	43	n/a	6%	7%	n/a
(Legro et al. 2014) [24]	Open-label, randomized trial		PCOS	Lifestyle vs oral contraceptive pills vs Combined before ovulation induction with clomiphene	149	50	49	50	14%	9%	28%
(De Paula Guedes Neto et al. 2011) [52]	Double-blind randomized trial		PCOS	Raloxifen vs Placebo	82	42	40	n/a	2%	3%	n/a
(Zahran et al. 2018) [53]	Open-label, randomized trial		PCOS	Clomiphene + Cabergolin vs Clomiphene	130	65	65	n/a	20%	12%	n/a
**MEN**											
**Author, Year**	**Study Design**		**Reason for Subfertility**	**Fertility Treatment and Protocol**	**Study population (N)**	**Group 1 (n)**	**Group 2 (n)**		**%** **headache in group 1**	**% headache in group 2**	
(Shuling et al. 2019) [54]	Prospective clinical trial		Idiopathic Oligozoospermia	Letrozole	15	n/a	n/a		25%	n/a	
(Ullur et al. 2025) [55]	Double-blind randomized trial		Oligoasthenoteratozoospermia	Letrozole vs Placebo	54	28	26		21%	n/a	
(Shah et al. 2015) [56]	Retrospective cohort study		Hypogonadism and with a BMI ≥ 25 kg/m2	Anastrozole	30	n/a	n/a		3%	n/a	
(Shah et al. 2021) [57]	Retrospective, observational study		Hypogonadism	Anastrozole	30	n/a	n/a		3%	n/a	
(Jones et al. 2023) [58]	Double-blind randomized trial		Obesity-associated hypogonadotropic hypogonadism	Leflutozole (low dose or medium dose vs high dose)	271	199	71		5%	6%	
(Alder et al. 2018) [59]	Retrospective, observational study		Hypoandrogenia, hyperestrogenemia	Clomiphene citrate and Anastrazol	51	n/a	n/a		2%	n/a	
(WHO, 1992) [60]	Double-blind randomized trial		Idiopathic impairment of semen quality	Clomiphene citrate vs Placebo	141	70	71		"Two men who were on clomiphene treatment, complained of visual disturbances, dizziness and headaches after 30 and 120 days of treatment respectively."		
(Chandrapal et al. 2016) [61]	Retrospective, observational study		Hypogonadism, Azoospermia or severe oligoospermia	Clomiphene citrate	77	n/a	n/a		1%	n/a	
(Soares et al. 2018) [62]	Double-blind randomized trial		Obesity-associated hypogonadism	Clomiphene citrate vs Placebo	78	39	39		0%	2%	

Across studies, letrozole shows inconsistent rates of headache, ranging from 1% to 41%, yet no consistent pattern emerges with dose or duration [41, 43, 44, 46, 48, 49, 63, 64]. For example, in a trial of 66 women comparing an extended low-dose protocol with a short double-dose regimen, headache was the most frequent but non-severe adverse event, occurring in 10% vs. 5% of participants [44]. Similarly, a study of 174 participants reported identical headache rates (18% in both arms) for 2.5 mg versus 5 mg letrozole used together with human menopausal gonadotropin (hMG) [43].

Anastrozole, another aromatase inhibitor, showed comparably high headache frequency: in a phase I dose-comparison study, 36% of participants experienced headaches [42].

In contrast, clomiphene-based protocols consistently demonstrated lower headache frequencies. A retrospective study of 109 women with PCOS found headache rates of 6% and 7% across two clomiphene regimens [51]. Another study evaluating 112 cycles reported similarly low rates (5% with clomiphene vs. 0% without) in an hCG-based protocol [50].

In a randomized trial of 82 women with PCOS, raloxifene also showed minimal headache incidence, with headache rates comparable to placebo (2% vs. 3%) [52].

Three comparative studies of letrozole versus clomiphene produced inconsistent findings. A double-blind randomized trial including 159 women reported similar overall rates of adverse events but did not specify headache frequencies. It was noted only that headache occurred in the letrozole group, whereas migraine was reported in the clomiphene group, with no more details [41]. A retrospective cohort of 92 participants found that clomiphene was associated with more frequent adverse events, including headache, than letrozole (41.9% vs 8.2%) [45]. Finally, an open-label trial with 100 participants observed only minimal differences in reported headaches in the clomiphene compared to the letrozole group (6% vs 4% respectively) [46].

Combination regimens also showed mixed results. Two additional trials comparing letrozole alone with letrozole plus clomiphene in women with PCOS found higher headache rates in the letrozole-alone arm (28% vs 41%, and 26% vs 29%) [48, 49]. In contrast, a randomized trial of 100 participants reported higher headache rates with the combination of letrozole and clomiphene compared with clomiphene alone (13% vs 5%) [47].

Among other add-on therapies, the combination of cabergoline and clomiphene nearly doubled headache frequency compared with clomiphene alone in a study of 130 women with PCOS [53].

Interestingly, six studies reported headaches in association with aromatase inhibitors in men [54–59]. In a randomized dose-comparison study of 271 men, letrozole caused headaches in 5% of participants, without evidence of a dose–response relationship [58]. Two trials evaluating letrozole for male factor infertility, including oligozoospermia (low sperm concentration) and oligoasthenoteratozoospermia (combined impairment of sperm concentration, motility, and morphology) reported headache among the most common adverse effects, affecting 25% [54] and 21% of participants [55], respectively.

Anastrozole, by contrast, was associated with a much lower headache incidence: in two retrospective studies, each evaluating 30 men, only 3% reported a minor headache [56, 57] and its combination with clomiphene showed low incidence (2%) [59].

In the three studies reporting about clomiphene in men as a therapy for infertility related to low testosterone, headache was reported at low rates in randomized, double-blind, controlled trials, ranging from 0% in a study on obesity-associated hypogonadism to 3% in a trial of idiopathic male infertility [60–62].

#### Gonadotropins

Based on regulatory labeling from the FDA and EMA, headache is commonly reported as an adverse effect across all gonadotropin preparations, including recombinant and urinary-derived FSH, hMG, LH, and hCG formulations [65–75].

In our systematic review, however, the 21 trials with gonadotropins in women reported variable headache rates between 0 and 15% (Table 2). In women, the literature comprised many interventional studies, including randomized controlled and open-label trials, as well as one small case series. Study populations were heterogeneous and included women with PCOS, unexplained infertility, clomiphene resistance, anovulatory infertility, and mixed infertility indications. Sample sizes ranged widely, from *n* = 4 to *n* = 1,390. In men, evidence was limited to three interventional studies, primarily evaluating hCG with or without FSH in the context of hypogonadotropic hypogonadism or impaired spermatogenesis.

**Table 2:** Gonadotropins and headache reports. FSH: follicle-stimulating hormone, GH: growth hormone, GnRH: gonadotropin-releasing hormone, hCG: human chorionic gonadotropin, hMG: human menopausal gonadotropin, LH: luteinizing hormone, OHSS: ovarian hyperstimulation syndrome, PCOS: polycystic ovary syndrome

**Medication Group**		**Drug**	**Clinical Trials**	**Post-marketing Research** **(Central Nervous System)**		**FDA-Label Report Link**					
Gonadotropin Recombinant FSH		Gonal-f	Headache 10.2%	Headache		https://www.accessdata.fda.gov/drugsatfda_docs/label/2020/020378s045,s067,s075lbl.pdf					
		Puregon (Follistim)	Headache 7.3%	Headache		https://www.accessdata.fda.gov/drugsatfda_docs/label/2011/021211s011lbl.pdf					
		Bemfola	Headache very common	Headache		https://www.ema.europa.eu/en/documents/product-information/bemfola-epar-product-information_en.pdf					
		Ovaleap	Headache very common	Headache		https://www.ema.europa.eu/en/documents/product-information/ovaleap-epar-product-information_en.pdf					
Gonadotropin Urinary FSH		Menopur	Headache 34.1%	Headache, dizziness		https://www.accessdata.fda.gov/drugsatfda_docs/label/2004/21663lbl.pdf					
		Bravelle	Headache 8.1–12.7% (IVF)	Headache		https://www.accessdata.fda.gov/drugsatfda_docs/label/2002/21484_Bravelle_lbl.pdf					
Gonadotropin hMG (FSH + LH)		Repronex	Headache 5.2% (s.c.) to 6.0% (i.m.)	Headache		https://www.accessdata.fda.gov/drugsatfda_docs/label/1999/21047lbl.pdf					
Gonadotropin LH Preparation		Luveris	Headache 9.9–10.2% (up do dosage)	Headache, fatigue		https://www.accessdata.fda.gov/drugsatfda_docs/label/2004/21322lbl.pdf					
hCG (urinary)		Pregnyl	n/a	Headache, depression, fatigue		https://www.accessdata.fda.gov/drugsatfda_docs/label/2011/017692s021lbl.pdf					
hCG (recombinant)		Ovitrelle/ Ovidrel (Choriogonadodropin alfa)	n/a	Headache		https://www.accessdata.fda.gov/drugsatfda_docs/label/2000/21149lbl.pdf					
		Novarel	n/a	Headache, depression, fatigue		https://www.accessdata.fda.gov/drugsatfda_docs/label/2011/017016s156lbl.pdf					
**WOMEN**											
**Author, Year**	**Study Design**		**Reason for Subfertility**	**Fertility Treatment and Protocol**	**Study population (N)**	**Group 1 (n)**	**Group 2 (n)**	**Group 3 (n)**	**%** **headache in group 1**	**% headache in group 2**	**%** **headache in group 3**
(Diamond et al. 2015) [76]	Randomized, controlled trial		Unexplained infertility	Gonatropin vs Clomiphene vs Letrozole	900	301	300	299	30.0%	34.9%	41.9%
(Norman et al. 2011) [77]	Phase III uncontrolled trial		Different reasons	FSH (rFSH)	682	682			9.1%		
(Recombinant Human FSH Study Group 1998) [78]	Open-label, randomized trial		Different reasons	FSH (rFSH vs uFSH)	127	60	63	n/a	15.0%	7.0%	n/a
(Dickey et al. 2002) [79]	Open-label, randomized trial		Different reasons	FSH (Urofollitropin s.c. vs. Urofollitropin i.m. vs Follitropin B)	177	60	59	58	8.3%	16.9%	10.2%
(Boostanfar et al. 2016) [80]	Double-blind, randomized,active-controlled trial		Different reasons	FSH (Corifollitropin alfa vs rFSH)	298	151	147	n/a	5.3%	3.4%	n/a
(Pasqualini et al. 2021) [81]	Randomized, single-blind trial		Different reasons	FSH (Folitime vs rFSH)	93	49	44	n/a	14.0%	4.5%	n/a
(Balen et al. 2007) [82]	Open-label, randomized trial		Anovulatory infertility and resistance to clomiphene citrate	FSH (rFSH vs FSH)	151	73	78	n/a	14.1%	13.7%	n/a
(Leader & Monofollicular Ovulation Induction Study, 2006) [83]	Randomized, group-comparative study		Anovulation or oligoovulation	FSH (Follitropin beta 25 IU vs 50 IU)	158	80	78	n/a	11.3%	9.0%	n/a
(Baldini et al. 2023) [84]	Case Series		Different reasons	FSH (Follitropin delta)	4	n/a	n/a	n/a	25.0%	n/a	n/a
(Boostanfar et al. 2015) [85]	Double-blind, randomized trial		Different reasons	FSH (Corifollitropin alpha vs rFSH)	1,390	695	696	n/a	6.1%	5.7%	n/a
(Strowitzki et al. 1995) [86]	Open-lable trial		Different reasons	FSH (rFSH vs FSH)	58	15	43	n/a	Headache as a side-effect (not specified)		
(Blockeel et al. 2022) [87]	Prospective observational study		Different reasons	FSH (Follitropin dellta)	944	893	n/a	n/a	0.2%	n/a	n/a
(Taketani et al. 2010) [88]	Single-blind, randomized trial		Oligo- or anovulatory infertility	FSH (Follitropin alpha vs urofollitropin)	261	129	132	n/a	4.7%	6.1%	n/a
(Humaidan et al. 2017) [89]	Randomized, single-blind trial		Poor ovarian responders	FSH (rFSH + LH vs rFSH)	939	477	462	n/a	6.1%	5.9%	n/a
(Marrs et al. 2004) [90]	Open-label, randomized trial		Male-factor (not specified)	FSH (rFSH vs rFSH +LH)	431	212	219	n/a	“The most common adverse events were headache and OHSS, each occurring in 18 patients.”		
(Nichols et al. 2001) [91]	Open-label, randomized trial		Premenopausal anovulatory and oligoovulatory infertility	FSH + LH s.c. vs FSH + LH i.m. vs hMG	108	36	36	36	6.0%	8.0%	19.0%
(Keye et al. 2004) [92]	Open-label, randomized trial		Different reasons	hMG (FSH vs hMG)	228	76	79	73	2.6%	8.9%	11.0%
(Witz et al. 2020) [93]	Single-blind, randomized trial		Different reasons	hMG vs FSH	619	310	309	n/a	9.4%	7.0%	n/a
(Platteau et al. 2006) [94]	Single-blind, randomized trial		Anovulatory infertility	hMG vs FSH	184	91	93	n/a	5.4%	6.5%	n/a
(Alviggi et al. 2007) [95]	Open-label, randomized trial		Unknown	hMG (s.c. vs i.m.)	168	85	83	n/a	“The recorded incidence of adverse effects was overall very low and similar in the two subgroups (2.4% vs. 3.7% of patients in the s.c. and i.m. subgroups, respectively); these adverse effects were headache, or aspecific abdominal pain, both not clearly related to the drug's intake."		
(European and Australian Multicenter Studygroup, 1995) [96]	Randomzied, placebo-controlled trial		Hypogonadotropic hypogonadism	GH (hMG+ GH vs hMG + Placebo)	64	48	16	n/a	2.0%	0.0%	n/a
(Dai et al. 2023) [63]	Open-label, randomized trial		PCOS	Letrozole vs Letrozole + hMG	174	87	87	n/a	31.0%	25.3%	n/a
**MEN**											
**Author, Year**	**Study Design**		**Reason for Subfertility**	**Fertility Treatment and Protocol**	**Study population (N)**	**Group 1 (n)**	**Group 2 (n)**		**%** **headache in group 1**	**% headache in group 2**	
(Nieschlag et al. 2017) [97]	Open-label trial		Hypogonadotropic hypogonadism	hCG	23	n/a	n/a		16.7%	n/a	
(Babak et al. 2018) [98]	Randomized, group-comparative study		Infertile patients with varicocele and an abnormal semen analysis	Varicocelectomy + hCG vs Varicocelectomy	193	94	99		1.0%	0.0%	
(Burgues & Calderon, 1997) [99]	Open-label trial		Hypogonadotropic hypogonadism	FSH + hCG	60	n/a	n/a		0.0%	n/a	

One study compared fertility treatment across clomiphene, letrozole, and gonadotropins. This large randomized trial of 900 women with unexplained infertility observed higher and more comparable rates: 35% in the clomiphene group, 42% in the letrozole group, and 35% among those treated with gonadotropins [76].

In trials of recombinant FSH formulations, including follitropin delta and corifollitropin alfa (a long-acting recombinant FSH analogue), headache occurred in 0.2%–15% of participants [77–88]. Studies comparing highly purified or urinary-derived FSH, such as urofollitropin, showed similar rates between 4% and 14% [79, 82, 88].

In a randomized trial including 939 participants, comparing FSH plus LH with FSH alone, headache was reported in 6% in each group. Another study with 431 participants comparing FSH plus LH with FSH alone identified headache as the most frequently reported adverse event, although the authors did not specify differences between treatment groups [89, 90].

In hMG-based regimens, either alone or in combination with recombinant FSH, headache occurred in 2%–10% of cases [91–96].

In contrast, a study of 174 women with PCOS comparing letrozole alone with letrozole plus hMG reported high headache rates (31% and 25%, respectively) suggesting that protocols involving aromatase inhibitors may be associated with more frequent headache than gonadotropin-add on regimens [63].

Among three studies involving men treated with hCG either combined with varicocelectomy or alongside FSH, headache occurred in isolated cases, though in one study the intensity was severe enough to prompt withdrawal [97–99].

#### GnRH Analogues

FDA-labeling identifies headache as one of the most frequently reported central nervous system adverse effects of both GnRH agonists and antagonists, with migraine explicitly noted in some post-marketing data [100–104]. In alignment with these data, 14 studies identified in this review likewise reported headache as a common adverse event during GnRH analogues treatment in women (Table 3) [64, 105–115]. Reported frequencies varied widely, ranging up to 45%, with a mean incidence of approximately 24%. Comparisons between GnRH agonists (buserelin, leuprorelin, triptorelin, goserelin, nafarelin) and antagonists (cetrorelix, ganirelix, relugolix, antide) revealed no consistent differences in headache occurrence. In a study of 95 women with PCOS randomized to three treatment groups, headache was reported in 0% of those receiving clomiphene, 12% across two GnRH agonist protocols, and 33% in the GnRH antagonist group [116].

**Table 3:** GnRH analogues and headache reports. FSH: follicle-stimulating hormone, GnRH: gonadotropin-releasing hormone, PCOS: polycystic ovary syndrome; SERM: selective estrogen receptor modulators

**Medication Group**		**DRUG**	**Clinical Trials**	**Post-marketing Research** **(Central Nervous System)**		**FDA-Label Report Link**					
GnRH Agonist		Leuprorelin (Lupron)	n/a	n/a		https://www.accessdata.fda.gov/drugsatfda_docs/label/2022/019732s045,020517s043lbl.pdf					
		Buserelin	Headache 63–75%	Headache, dizziness, nervousness, migraine (4–7%)		https://www.accessdata.fda.gov/drugsatfda_docs/label/2023/019726s069lbl.pdf					
		Nafarelin	n/a	Headache		https://www.accessdata.fda.gov/drugsatfda_docs/label/2012/019886s030lbl.pdf					
GnRH Antagonist		Cetrorelix (Cetrotide)	Headache 1.1%	Headache		https://www.accessdata.fda.gov/drugsatfda_docs/label/2008/021197s010lbl.pdf					
		Ganirelix (Orgalutran)	Headache 24%	Headache		https://www.accessdata.fda.gov/drugsatfda_docs/label/2014/021057s010lbl.pdf					
**WOMEN**											
**Author, Year**	**Study Design**		**Reason for Subfertility**	**Fertility Treatment and Protocol**	**Study population (N)**	**Group 1 (n)**	**Group 2 (n)**	**Group 3 (n)**	**%** **headache in group 1**	**% headache in group 2**	**%** **headache in group 3**
(Prajapati et al. 2017) [116]	Prospective, observational study		PCOS	Clomiphene vs GnRH agonist (long and short) vs GnRH Antagonist	95	38	51	6	0.0%	11.8%	33.3%
(Cozzolino et al. 2023) [64]	Prospective observational study/case reports		Adenomyosis	GnRH-Agonist and Letrozole	4	n/a	n/a	n/a	one case presented with migraine (not specified whether they were pre-existing)		
(El-Nemr et al. 2002) [105]	Comparative cohort study		Different reasons	Buserelin vs Leuprorelin vs Nafarelin	157	51	53	53	37.3%	45.3%	60.4%
(J.A. Huirne et al. 2006) [106]	Randomized study		Different reasons	Cetrorelix vs Buserelin	182	91	91	n/a	17.6%	40.7%	n/a
(Amir et al. 2005) [117]	Retrospective study		Different reasons	different	98	98	n/a	n/a	28.6%		
(Huirne et al. 2004) [107]	Double-blind, randomized trial with open-label treatment phase		Different reasons	GnRH Antide	144	144	n/a	n/a	“most frequently recorded side effects were general disorders (especially fatigue and headache)”		
(Judith Af Huirne et al. 2006) [108]	Randomized trial		Different reasons	GnRH Antide	63	31	32	n/a	37.0%	45.0%	n/a
(Tapanainen et al. 1993) [109]	Open-label, randomized trial		Different reasons	Goserelin vs Buserelin	100	49	51	n/a	“ … patients in the buserelin group suffered more from headache than those given s.c. goserelin.”		
(Sauer et al. 2004) [110]	Open-label, randomized trial		Different reasons	Leuprolide vs Certorelix	74	74	n/a	n/a	“ … 17 (23.0%) reported adverse event” (not specified)		
(Goldman et al. 1994) [111]	Randomized trial		Different reasons	Nafarelin vs Buserelin	108	53	55	n/a	18.9%	12.7%	n/a
(Lockwood 1995) [115]	Randomized, single-blind trial		Different reasons	Nafarelin vs Buserelin	342	172	170	n/a	19.0%	50.0%	n/a
(Komiya et al. 2022) [112]	Open-label, case–control trial		Different reasons	Relugolix vs Ganirelix	785	127	658	n/a	0.0%	1.6%	n/a
(Simons et al. 2005) [113]	Double-blind, randomized trial		Different reasons	Triptorelin (different protocol length: short medium and long)	178	58	62	58	3.4%	4.8%	0.0%
(Lobo et al. 2024) [114]	Open-label, randomized trial		Different reasons	Triptorelin vs Cetrorelix	437	221	216	n/a	13.4%	14.2%	n/a
**MEN**											
**Author, Year**	**Study Design**		**Reason for Subfertility**	**Fertility Treatment and Protocol**	**Study population (N)**	**Group 1 (n)**	**Group 2 (n)**	**Group 3 (n)**	**%** **headache in group 1**	**% headache in group 2**	**%** **headache in group 3**
(Foresta et al. 2004) [118]	Randomized, group-comparative study		Oligozoospermia	Leuprolide + r-hFSH vs r-hFSH	97	n/a	n/a	n/a	16.0%	0.0%	n/a

In a trial of 97 men treated with either a combination of the GnRH agonist leuprolide acetate plus FSH or FSH alone, headaches were only reported during the GnRH-agonist phase, affecting 16% of participants [118].

#### Luteal Phase Support

FDA-Drug label data indicate that estrogen and progesterone formulations used for luteal support frequently list headache and migraine among central nervous system–related adverse effects [119–123].

Compared to the FDA-Data, we found in ten studies evaluating estradiol and progesterone preparations for luteal support that headache was reported inconsistently (Table 4). In studies using estradiol (oral, transdermal, or gel formulations), headache occurred in 0.6%–17.3% of participants [124–126]. Comparisons between oral and transdermal estradiol indicated a slightly higher incidence of headache with oral administration, while also the plasma levels of estradiol are higher with oral administration. Interestingly, in a cross-over trial of 34 women comparing clomiphene plus placebo with clomiphene plus estradiol for effects on endometrial thickness, three headache events were reported in the placebo group, two of which led to discontinuation, whereas no headache occurred in the estradiol group [126].

**Table 4:** Luteal support and headache reports

**Medication Group**		**Drug**	**Clinical Trials**	**Post-marketing Research** **(Central Nervous System)**		**FDA-Label Report Link**					
Progesterone (vaginal)		Crinone gel	Headache 13–17%	Headache, dizziness,depression, nervousness		https://www.accessdata.fda.gov/drugsatfda_docs/label/2013/020701s026lbl.pdf					
Progesterone (oral)		Dydrogesterone (Duphaston)	Common	Migrane/headaches, dizziness, Somnolence		https://verification.fda.gov.ph/files/DRP-221_PI_01.pdf					
Progesterone (IM)		Progesterone in oil	n/a	Headache, fatigue, nervousness, dizziness		https://www.accessdata.fda.gov/drugsatfda_docs/label/2007/017362s104lbl.pdf					
Estrogen		Estradiol valerate (Delestrogen)	n/a	Headache, migraine, dizziness, depression, nervousness		https://www.accessdata.fda.gov/drugsatfda_docs/label/2017/009402s052lbl.pdf					
Estrogen		Estrogen (oral/transdermal)	Headache 13–14% depending on dose	Headache, migraine, dizziness, depression, nervousness		https://www.accessdata.fda.gov/drugsatfda_docs/label/2025/004782s179lbl.pdf					
**WOMEN**											
**Author, Year**	**Study Design**		**Reason for Subfertility**	**Fertility Treatment and Protocol**	**Study population (N)**	**Group 1 (n)**	**Group 2 (n)**	**Group 3 (n)**	**%** **headache in group 1**	**% headache in group 2**	**%** **headache in group 3**
(Garimella et al. 2021) [124]	Prospective, observational trial		Different reasons	Estradiol (oral vs transdermal)	294	156	138		17.3%	3.6%	
(Tran et al. 2024) [125]	Open-label, randomized trial		Different reasons	Estradiol (oral vs transdermal)	380	190	190		5.5%	0.6%	
(Satirapod et al. 2014) [126]	Double-blind, cross-over, placebo-controlled trial		Healthy women	Estradiol (oral) + Clomiphene vs Clomiphene + Placebo	34	30	30		0.0%	10.0%	
(Colombo et al. 2023) [127]	Randomized controlled trial		Different reasons	Progesterone vs placebo	470	235	235		44.3%	45.6%	
(Karadeniz et al. 2023) [128]	Randomized controlled trial		Different reasons	Progesterone (intramuscular vs vaginal vs oral)	195	50	94	51	2%	0%	15.7%
(Ozer et al. 2021) [129]	Open-label, randomized trial		Different reasons	Progesterone (oral vs trans vaginal)	134	67	67		13.8%	0.0%	
(Lockwood et al. 2014) [130]	Open-label, randomized trial		Different reasons	Progesteron (subcutaneous vs. Gel)	683	339	344		5.3%	4.9%	
(Stadtmauer et al. 2013) [131]	Prospective, randomized, single-blind, phase III clinical trial		Different reasons	Progesterone (vaginal ring vs vaginal gel)	1,297	646	651		20.0%	26.0%	
(Bergh et al. 2012) [132]	Assessor-blinded, randomized trial		Different reasons	Progesterone (Gel bs tablets)	2,057	991	992		1/6 cases of adverse events was due to headache (no specification)		
(Ng et al. 2003) [133]	Open-label, randomized trial		Different reasons	Progesterone (Tablet vs gel)	60	30	30		“No significant differences were demonstrated in ( …)headache.”		

Seven trials investigating progesterone (including vaginal, oral and injectable formulations) reported variable rates between 0% and 20% [127–133].

### Systematic Assessment of Headache Data Across Common Fertility Treatments

The only study that systematically assessed headache patterns in the context of fertility treatment, a retrospective telephone-based survey of 98 women, found that headaches occurred significantly more often during the initial GnRH analogue treatment for pituitary downregulation phase of IVF, although the specific agent used was not reported [117]. Notably, 17.8% of non-migraine patients also reported IVF-related headaches.

Across all other fertility treatment studies included in this review, reporting of headache was markedly nonspecific. Only one study differentiated between migraine and headache, but also with no further specification and no incidence rate, and none documented whether participants had pre-existing headache or migraine [41]. Headache was only reported as a general adverse event, without details on severity, timing, or clinical phenotype.

### Headache and Treatment-Related Complications

Complications arising during or after fertility treatment are of particular interest when evaluating the occurrence of headache (Table 5). Across 15 case reports and one retrospective case-control study [134–149] headache mostly preceded neurological deficits and often occurred in the context of OHSS. Vascular events were the most common, including cerebral venous thrombosis, ischemic stroke, and reversible cerebral vasoconstriction syndrome, typically arising during or shortly after ovarian stimulation. However, also one male patient developed intracranial venous thrombosis after clomiphene use [136].

**Table 5:** Treatment complications. GnRH: gonadotropin-releasing hormone, IVF: In-vitro fertilization, PCOS: polycystic ovary syndrome; SERM: selective estrogen receptor modulators

Author, Year	Titel	Study Design	Reason for Infertility	Fertility treatment	Phase	Category
(Man & Hui, 2011) [134]	Cerebral venous thrombosis secondary to ovarian hyperstimulation syndrome	Case Report	Unknown	Full Protocol	Ovarian hyperstimulation	vascular
(Akinci & Anash, 2025) [135]	Cytotoxic Lesion of the Corpus Callosum Related to Migraine With Aura Triggered by In Vitro Fertilization and Embryo Transfer: A Case Report.	Case Report	PCOS	Full Protocol	10 days after fertility treatment and no pregnancy	vascular
(Zahid et al. 2016) [136]	Intracranial venous thrombosis in a man taking clomiphene citrate.	Case Report	Oligospermia	SERM (Clomiphene)	Male infertility	vascular
(Koh et al. 2021) [137]	Ischemic Stroke Associated With Ovarian Hyperstimulation Syndrome	Case Report	Unknown	Full Protocol	Ovarian hyperstimulation	vascular
(Tehraninejad et al. 2010) [138]	Late onset fasting triggered thrombosis of internal carotid artery after ovarian stimulation	Case Report	PCOS	Full Protocol	Ovarian stimulation	vascular
(Akça & Özdemir, 2022) [139]	Postpartum intracerebral hematoma following in vitro fertilization	Case Report	Unknown	Full Protocol	Post-partum	vascular
(Shmorgun et al. 2009) [140]	Renal artery dissection during an in vitro fertilization/intracytoplasmic sperm injection cycle	Case Report	Secondary infertility (not specified)	Full Protocol	Ovarian stimulation	vascular
(Freilinger et al. 2010) [141]	Reversible cerebral vasoconstriction syndrome associated with hormone therapy for intrauterine insemination	Case Report	Unexplained infertility	Full Protocol	After insemination	vascular
(Kobak et al. 2014) [142]	Scleroderma renal crisis and ovarian hyperstimulation syndrome related to the use of clomiphene in a patient with scleroderma	Case Report	Unknown	SERM (Clomiphene)	Ovarian hyperstimulation	vascular
(Edris et al. 2007) [143]	Successful management of an extensive intracranial sinus thrombosis in a patient undergoing IVF: case report and review of literature.	Case Report	Multiple uterine fibroid	Full Protocol	Ovarian hyperstimulation	vascular
(Motegi et al. 2012) [144]	Hemorrhagic onset of rhabdoid meningioma after initiating treatment for infertility.	Case Report	Unknown	SERM (Clomiphene)	Ovarian stimulation	tumor
(Ramos et al. 2022) [145]	Meningeal Melanomatosis with a Spinal Meningeal Melanocytoma Trigger by an in vitro Fertilization	Case Report	Unknown	Full Protocol	Unspecific “after IVF” Treatment	tumor
(Alnahas et al. 2025) [146]	Pituitary Mimic: Sellar Meningioma in a Patient Undergoing Fertility Therapy.	Case Report	Hypertension, prediabetes, hypothyroidism, and mild hyperprolactinemia	GNRH-Agonist (unspecific)	Ovarian stimulation	tumor
(Gaul et al. 2007) [147]	Cluster headache triggered by high-dose gestagens in the context of in vitro fertilization: a case report.	Case Report	Endometriosis and male subfertility	Full Protocol	Luteal support	primary headache
(Rollene et al. 2011) [148]	Migraines and ovarian hyperstimulation syndrome: a dopamine connection.	Retrospective case-control study	Unknown	Full Protocol	Ovarian hyperstimulation syndrom	primary headache
(Rodrigues et al. 2014) [149]	Psychotic episode secondary to gonadotrophins	Case Report	Secondary infertility (not specified)	Full Protocol	Ovarian stimulation	other

Other rare complications included intracerebral hematoma after IVF and cesarean delivery [139] and tumor-related events such as meningioma or melanomatosis following gonadotropin or clomiphene therapy [144–146]. One case described a psychotic episode with severe headache secondary to gonadotropin administration [149].

While most cases described secondary headache due to identifiable complications, three publications focused on the onset or exacerbation of primary headache disorders in relation to hormonal therapy.

Rollene et al (2011) conducted a retrospective case–control study comparing women who developed OHSS after IVF with those who did not [148]. A prior history of migraine was a strong predictor for developing OHSS. In the univariate analysis (combined cohort of IVF and superovulation), the risk of OHSS was increased 4.8-fold in patients with a history of migraine (OR = 4.78, 95% CI 2.63–8.66, *p* < 0.001).

One report described a 26-year-old woman who developed migraine with aura 1.5 years before the reported complication, at the time she began fertility treatment for PCOS [135]. The onset of migraine with aura in early adulthood is common and does not necessarily indicate a causal relationship with the underlying reproductive condition. The most severe migraine attack occurred shortly after a failed embryo transfer, when MRI revealed a cytotoxic lesion of the corpus callosum. This lesion type is believed to result from inflammatory cascades leading to cytokine release and oxidative stress, ultimately causing cytotoxic edema.

Gaul et al (2007) reported the case of a woman with no prior headache history who developed a headache fulfilling the ICHD-II criteria for episodic cluster headache during luteal phase support with high-dose progesterone [147]. The attacks ceased upon discontinuation of the treatment.

### Psychosocial Aspects and Headache During Fertility Treatment

Only six studies retrieved within our systematic search also assessed psychosocial aspects in the context of headache and fertility treatments, providing preliminary insights.

Five studies investigated pharmacological influence on mental well-being in women undergoing fertility treatment. Two observational studies reported that progesterone, particularly with oral administration [128] and menotropin [150] were associated not only with headache but also with mood-related effects, such as depression or mood changes. Similarly, a randomized comparative study found that intranasal GnRH agonists were linked to both higher rates of headache and depression than subcutaneous administration, despite comparable efficacy and higher plasma levels with subcutaneous administration [151]. In contrast, no differences in psychosocial wellbeing or headache were observed between immediate versus postponed modified natural cycle frozen-thawed embryo transfer, or between treatment with or without vaginal progesterone, although high baseline stress was common [127, 152].

A population-based survey further underscored the role of contextual influences: women in fertility clinics were more likely to live in urban areas, to have private insurance, and to undergo IVF, whereas community-based women often used hormonal treatment or none. Notably, clinic patients reported fewer headaches and less fatigue but higher rates of anxiety disorders [153].

## Discussion

This systematic review demonstrates that headache is a frequent but inconsistently reported adverse event across fertility treatments. Available evidence is highly heterogeneous, with multiple treatment classes, dosing protocols, and study populations. Headache rates ranged from rare (<1%) to nearly 50%, depending on the agent and design. Although the FDA and EMA labeling of fertility drugs frequently lists headache, and in some cases migraine as a common adverse event, the clinical trial literature found in our search using the specific search term “headache” remains superficial and inconsistent in its reporting. Almost no study provided standardized or detailed reporting on headache type, severity, or temporal association with treatment phases. Given that headache and migraine are highly prevalent in reproductive-age women, the observed rates in clinical studies likely underestimate the true burden [154].

Several mechanisms may help explain why aromatase inhibitors and GnRH analogues show high headache frequencies in fertility treatment. The action of GnRH analogues is time-dependent: while an initial pulsed or short-term administration produces a transient rise in LH, FSH, and estrogen, continuous administration over several weeks leads to downregulation of pituitary GnRH receptors, resulting in a marked decline in LH, FSH, and ultimately estrogen levels [25]. This estrogen suppression parallels the pharmacological effect of aromatase inhibitors such as letrozole, which also induce rapid and profound reductions in circulating estrogen [155].

The retrospective study by Amir et al (2005) supports this mechanistic link, showing that headaches occurred most frequently during the initial GnRH analogue treatment for pituitary downregulation and again after unsuccessful cycles, another period characterized by estrogen decline [117]. Notably, even women without migraine reported headaches during these phases, suggesting that abrupt hormonal shifts can lower the threshold for headache initiation.

Estrogen withdrawal is a well-established trigger of migraine and may increase susceptibility by modulating trigeminovascular activation, altering serotonergic and CGRP pathways, and influencing vascular reactivity [156].

Gonadotropins have been shown to act directly on endothelial cells through their respective receptors, stimulating endothelial nitric oxide synthase activity and nitric oxide release, which promotes vasodilation. At elevated concentrations, however, FSH may impair endothelial barrier function by altering vascular endothelial cadherin distribution and increasing vascular permeability, potentially compromising vascular stability and contributing to vascular risk [157]. Theories linking endothelial dysfunction and vascular instability may also explain overlapping phenomena between headache and OHSS. Indeed, one retrospective study demonstrated a nearly fivefold increased risk of OHSS among women with a history of migraine, supporting a shared vascular susceptibility, thus underlying causality remains uncertain [148].

Although this review on headache reports in the context of fertility treatment is focused on women, assessing headache in men undergoing treatment is equally important. Male infertility cases involve hypogonadism, a condition characterized by low testosterone production or impaired spermatogenesis. Relative androgen deficiency, especially when accompanied by elevated estradiol levels, has been linked to increased migraine susceptibility in men [158]. Hormone-modulating treatments used in male infertility can shift androgen–estrogen balance and may further influence headache threshold.

Given these endocrine dynamics, men with underlying hypogonadism or altered sex-hormone profiles may be particularly vulnerable to headache or migraine exacerbation during treatment. Monitoring headache in this population is therefore critical, not only to recognize potential adverse effects but also to better understand how sex-hormone regulation influences migraine biology in men [159].

In this systematic review, reports of tumor-related events were limited to isolated case reports. In these cases, a temporal association with fertility treatment was described. However, information on treatment duration and baseline status, including the presence of pre-existing lesions prior to treatment initiation, was often incomplete or unavailable. Accordingly, a causal relationship between fertility treatment and tumor development cannot be established, and these findings should be interpreted with caution.

This review is limited by substantial heterogeneity among the included studies, particularly regarding study design, population characteristics, and treatment regimens. Definitions of headache and reporting standards varied widely, introducing potential reporting bias. Headache as adverse events were typically reported in a non-specific manner, without details regarding its frequency, intensity, or temporal relationship to treatment. The literature search was conducted in two major biomedical databases, PubMed and Embase, which together provide extensive and complementary coverage of clinical, epidemiological, and pharmacological research. Given the emerging nature of this research field, this database selection was considered sufficient to capture the core body of relevant literature. In addition, the FDA and/or EMA drug labelling were manually screened. Nevertheless, it cannot be excluded that relevant publications indexed exclusively in other databases may have been missed, and this should be considered when interpreting the findings.

## Conclusion

Available evidence suggests that headache is a clinically relevant but inconsistently reported adverse outcome during fertility treatments. For clinicians, awareness of headache as a side effect of fertility treatment is crucial. Headache may occur in association with hormonal fluctuations, vasoactive effects, or as a manifestation of secondary complications such as OHSS or cerebrovascular events. Individuals with a pre-existing history of migraine may be at increased risk of severe headache during treatment. However, the current evidence is limited and heterogeneous.

The principal value of this systematic review lies in explicitly addressing an underexplored and frequently overlooked clinical outcome in assisted reproduction and in highlighting the paucity of standardized data on headache outcomes. Future trials should systematically evaluate headache, report timing, intensity, and headache type, and consider hormonal, vascular, and psychosocial mediators. Integrating patient-reported outcomes would enhance understanding of the clinical burden of headache in assisted reproduction.

## Data Availability

Data are provided within the manuscript or supplementary files.
